# Endovascular treatment of pseudoaneurysms secondary to chronic pancreatitis: reports of two cases

**DOI:** 10.1590/1677-5449.012517

**Published:** 2018

**Authors:** Fabiana Seifert Santos, Karolaine Marcelina da Silva Sousa, Thiago Augusto Cadorin de Castro, Felipe Coelho, Rodrigo Gomes de Oliveira, Walter Jr. Boim de Araujo, Lilian Cabral Pereira dos Santos, Raquel Canzi Almada de Souza

**Affiliations:** 1 Pontifícia Universidade Católica do Paraná – PUCPR, Curso de Medicina, Londrina, PR, Brasil.; 2 Santa Casa de Misericórdia de Londrina, Cirurgia Vascular, Londrina, PR, Brasil.; 3 Universidade de Brasília – UnB, Programa de Pós-graduação em Ciências Médicas, Brasília, DF, Brasil.; 4 Pontifícia Universidade Católica do Paraná – PUCPR, Londrina, PR, Brasil.; 5 Universidade Federal do Paraná – UFPR, Hospital de Clínicas, Serviço de Angiorradiologia e Cirurgia Endovascular, Curitiba, PR, Brasil.; 6 Universidade Federal do Paraná – UFPR, Hospital de Clínicas, Gastroenterologia, Curitiba, PR, Brasil.; 7 Universidade Federal do Paraná – UFPR, Hospital de Clínicas, Serviço de Endoscopia Digestiva, Departamento de Medicina Interna, Curitiba, PR, Brasil.

**Keywords:** pseudoaneurysm of the splenic artery, chronic pancreatitis, alcoholic pancreatitis, arteriography, endovascular surgery, pseudoaneurisma de artéria esplênica, pancreatite crônica, pancreatite alcoólica, arteriografia, cirurgia endovascular

## Abstract

Pseudoaneurysm secondary to chronic pancreatitis is a rare complication, but one with a high mortality rate. It is etiologically associated with chronic pancreatitis, and most diagnoses are made after rupture, which manifests with clinical signs of acute hemorrhage. Computed tomography plays an important role in diagnosis, but digital subtraction angiography remains the gold-standard method for diagnostic confirmation and for treatment planning. This article describes two cases of pseudoaneurysm in patients with chronic alcoholic pancreatitis; one involving the splenic artery and the other the gastroduodenal artery, complicated by thoracic and abdominal bleeding respectively. Both were successfully treated, using minimally invasive endovascular methods to implant coils and stent-grafts.

## Resumo

O pseudoaneurisma decorrente de pancreatite crônica consiste em complicação rara, porém com alta taxa de mortalidade. Está etiologicamente associado à pancreatite crônica, e seu diagnóstico é feito mais comumente após ruptura, manifestando-se através de sinais clínicos de hemorragia aguda. A tomografia computadorizada desempenha papel importante no diagnóstico; contudo, a angiografia por subtração digital mantém-se como método padrão-ouro para confirmação diagnóstica e direcionamento do tratamento. O presente artigo relata dois casos de pseudoaneurisma em pacientes com pancreatite crônica alcoólica, sendo um da artéria esplênica e outro da artéria gastroduodenal, complicados com sangramento torácico e abdominal respectivamente. Ambos foram submetidos a tratamento endovascular minimamente invasivo com sucesso, através de implante de molas e de *stent-grafts*.

## INTRODUCTION

 Chronic pancreatitis is a clinical condition that manifests with persistent or intermittent abdominal pains associated with inflammation of the pancreas and evidence of pancreas-related exocrine or endocrine insufficiency, such as diabetes and steatorrhea. Anatomopathological analysis reveals evidence of irreversible destruction of pancreatic tissue, with proliferation of dense collagen and fibroblasts in the parenchyma. 1

 The strong association between chronic pancreatitis and excessive alcohol consumption has been known since 1878, when Edmondson described the disease as “drunkard’s pancreas”. 2

 Pseudoaneurysms of the splenic artery are less frequent than true aneurysms of this artery. Fewer than 200 cases have been described in the literature 3 and they are usually associated with trauma and, primarily, with inflammatory processes in the abdominal cavity, such as pancreatitis. 4

 In contrast, true aneurysms of the splenic artery account for more than half of all visceral artery aneurysms. 5 Risk factors for true splenic artery aneurysm are hypertension, atherosclerosis, cirrhosis, portal hypertension, liver transplantation, female sex, pregnancy, and multiparity, 6 but can also include splenomegaly, medial fibrodysplasia, arteritis, collagen vascular disease, polyarteritis nodosa, systemic lupus erythematosus, anomalous splenic artery origin, a 1-antitrypsin deficiency, and inflammatory and infectious diseases. 5 ^-^ 7

 Although rare, SAAs can potentially be life threatening, due to spontaneous intraperitoneal rupture, rupture into the neighboring hollow organs, and fistulization into the pancreatic duct. 8

 The objective of this report is to describe two cases of pseudoaneurysm in patients with chronic alcoholic pancreatitis, one involving the splenic artery and the other the gastroduodenal artery and complicated by thoracic and abdominal bleeding respectively. Both were managed with minimally invasive endovascular treatment. 

## CASE REPORTS

### Case 1

 A 56-year-old, black, male patient was brought to hospital by the emergency services and admitted to the thoracic service after presenting with acute respiratory failure of unknown cause. He complained of discomfort in the left hemithorax, dry coughing and dyspnea. 

 He had a history of more than 20 years of alcoholism (spirits) and smoking (1 pack a day for 30 years). 

He reported arterial hypertension under control and no other comorbidities.

 Physical examination revealed that the patient was in good general health, with good color, good oxygenation, and free from fever. Cardiac auscultation was unremarkable. Pulmonary auscultation revealed rales in the left hemithorax and vesicular murmur was reduced at the ipsilateral base. Extremities exhibited good perfusion and pulses were strong and symmetrical in all limbs. 

 Thoracentesis was performed, followed by thoracostomy with closed left pleural drainage via underwater seal, because of high volume hemothorax. 

 The chest drain was removed after 5 days under observation and on the eleventh day in hospital the patient underwent pleuroscopy, which showed residual fluid with traces of blood. On the 12th day he was discharged from hospital with instructions to return for outpatients follow-up at the thoracic surgery service. 

 Thirty days after hospital discharge, the patient was referred for investigation by the vascular surgery team because of a pulsating swelling in the upper abdomen, with clinical suspicion of a thoracoabdominal aneurysm. 

**Figure 1:**
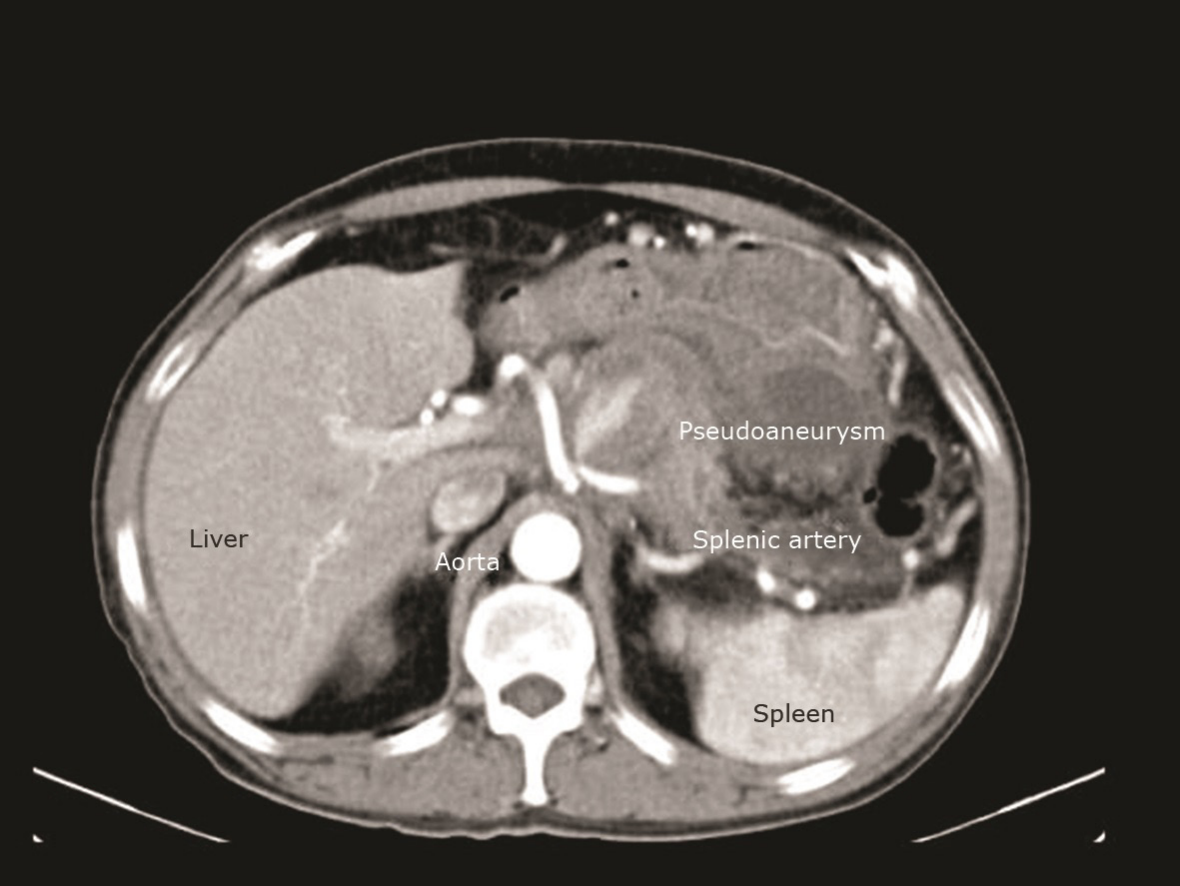
Computed tomography showing pseudoaneurysm of the splenic artery.

 Angiotomography of the chest, abdomen, and pelvis was conducted, ruling out thoracoabdominal aneurysm and raising a diagnostic hypothesis of pseudoaneurysm of the splenic artery, as shown in Figure 1 . Tumoral markers ruled out a diagnosis of pancreatic cancer and high lipase levels confirmed the diagnosis of pancreatitis. 

 In view of the suspected pseudoaneurysm of the splenic artery, the decision was taken to conduct digital subtraction angiography with selective catheterization of the celiac trunk, which confirmed the diagnosis of pseudoaneurysm of the splenic artery. 

**Figure 2:**
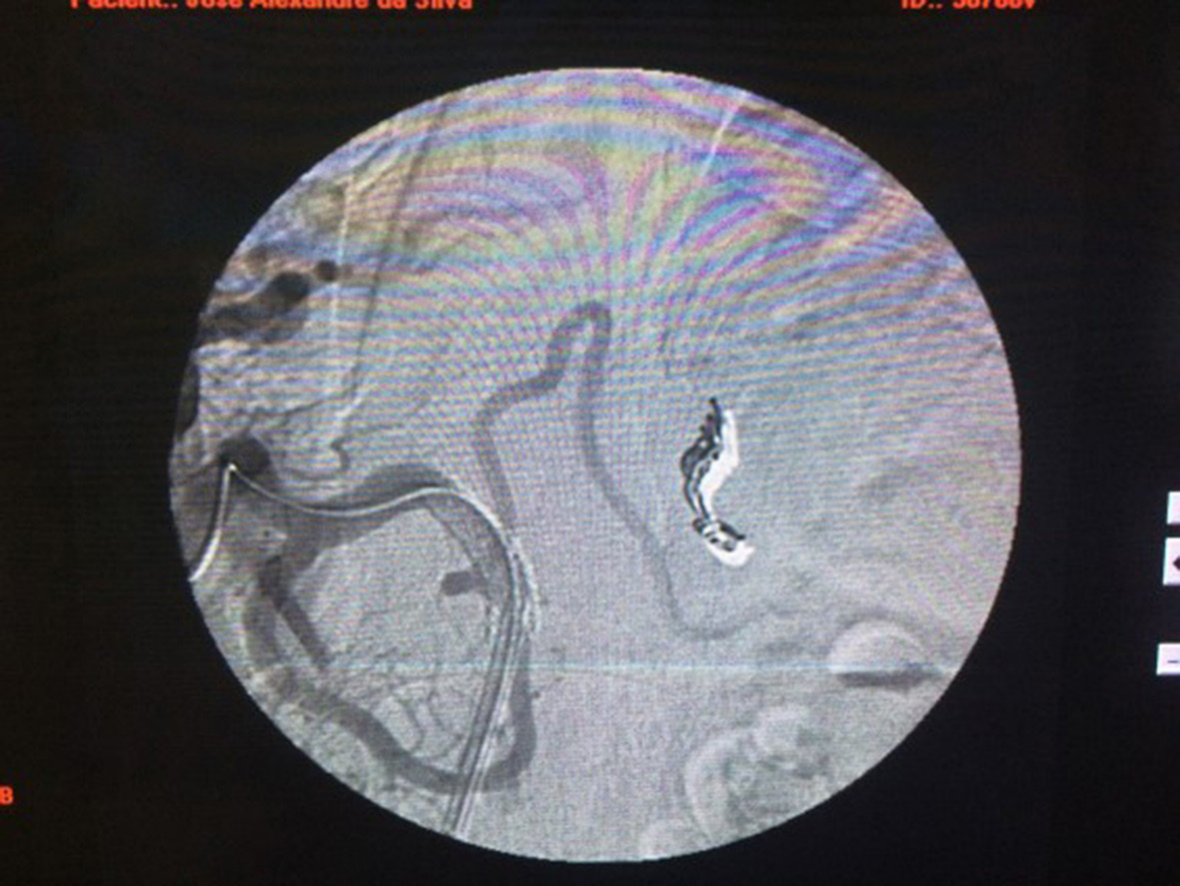
Arteriography showing pseudoaneurysm of the splenic artery.

 Having made this diagnosis, it was decided to treat the lesion using endovascular techniques. Under local anesthesia and venous sedation, femoral access was obtained via the right lower limb, a 0.035 guidewire was advanced via the celiac trunk and splenic artery, and three 8 × 50 mm controlled detachment coils were released, followed by placement of a 6 × 50 mm balloon-expandable covered stent, with coverage from the celiac trunk to the common hepatic artery, with the objective of excluding the splenic artery. This procedure was executed with no intercurrent conditions, and the final angiographic image can be seen in Figure 2 . 

 On the fifth day after the operation, the patient was vaccinated against encapsulated bacteria and discharged from hospital for outpatients follow-up. 

### Case 2

**Figure 3:**
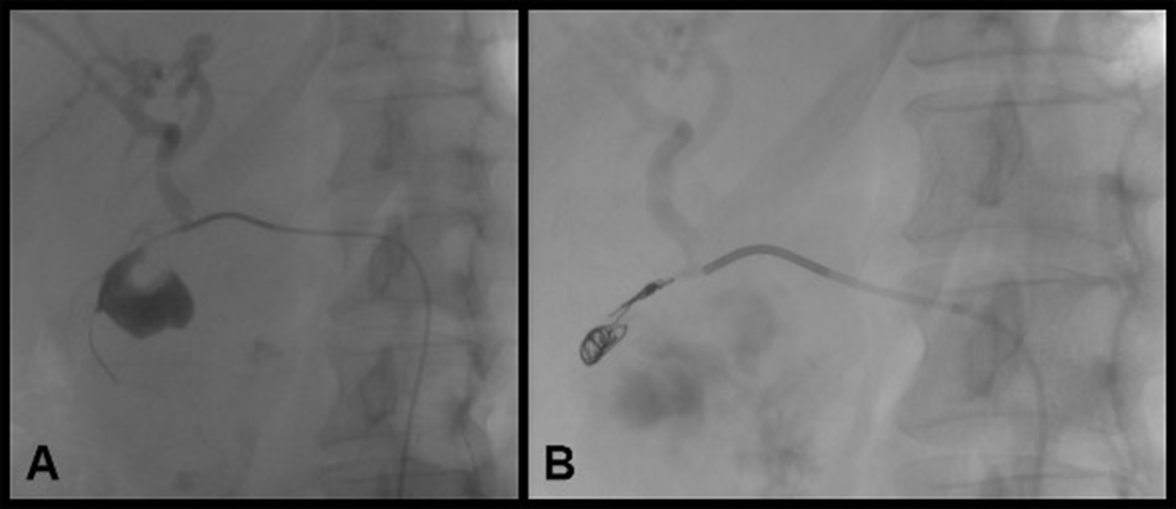
(A) Arteriography showing pseudoaneurysm of the gastroduodenal artery; (B) Control arteriography after embolization with microcoils.

 The patient was a 54-year-old female, with a history of chronic alcoholism, who suffered abdominal pains in the right flank, with sudden onset 5 months previously, associated with itching (pruritus), jaundice, choluria, and fecal acholia, and had lost 6 kg over the previous 3 weeks. Three days before hospital admission she had had an episode of fever that reached 38.5 °C. Total bilirubin was 12.9 mg/dl (with direct bilirubin of 9.7), alkaline phosphatase of 385 U/L and GGT of 149 U/L. She had presented with a similar condition 3 years previously, but had abandoned the investigation after spontaneous recovery. She was admitted to hospital to treat cholangitis and for investigation, where the chronic pancreatitis became acute and she began to vomit blood copiously. An upper digestive endoscopy showed esophageal varices and considerable swelling of the anterior wall of the duodenal bulb and papilla, where there were also dark biliary secretions (indistinguishable from bloody secretions). Abdominal palpation revealed a large pulsating swelling on the right flank. The patient was examined with computed tomography angiography of the abdomen, which identified a large pseudoaneurysm of the gastroduodenal artery. The tomographic findings were confirmed with digital subtraction angiography and the decision was taken to embolize the gastroduodenal artery with controlled detachment coils, as an essential measure to control the active bleeding. As illustrated in Figure 3 , the procedure was successful and enabled clinical and hemodynamic stabilization of the patient, after volume resuscitation and blood transfusion. 

## DISCUSSION

 Asymptomatic pseudoaneurysms of the splenic artery can become medical emergencies because of rupture and bleeding, although diagnosis is generally incidental and made before rupture occurs. They are frequent complications, particularly since endovascular procedures have become popular. 

 There are asymptomatic cases reported in the medical literature. However, pseudoaneurysms more commonly manifest through silent anemia, melena, pain in the upper left quadrant, or copious bleeding followed by hemodynamic instability. 4 ^,^ 5 Therefore, a pseudoaneurysm should be considered a disease requiring emergency care that must be diagnosed and treated promptly and precisely. 9 ^,^ 10

 The splenic artery and other branches that originate from the celiac trunk have an intimate relationship with the pancreas and are the vessels most commonly involved in severe acute pancreatitis and in chronic pancreatitis. 11 ^,^ 12

 Pseudoaneurysms are considered one of the most severe manifestations of a vast spectrum of complications of pancreatitis. Their locations are directly related to the distribution of pseudocysts or other accumulations of liquid seen in pancreatitis. 13 It is believed that the mechanism of pseudoaneurysm formation is triggered by “digestion” of the artery wall by pancreatic enzymes. 3

 Around 50% of pseudoaneurysms of the splenic artery are associated with pancreatitis, while another significant proportion are correlated with abdominal traumas. 3 ^,^ 14 ^-^ 16 Depending on clinical presentation, pseudoaneurysms of the splenic and gastroduodenal arteries can be diagnosed by ultrasound with Doppler, angiotomography, or digital subtraction angiography, the last of which is considered the gold-standard method. 3

 Although there are descriptions in the literature of conservative treatment of pseudoaneurysms of the splenic artery and the gastroduodenal artery, the majority of authors recommend surgical treatment, in order to prevent hemorrhagic complications caused by rupture of the pseudoaneurysm. 3 Patients admitted via the emergency room with hemodynamic instability can represent a challenge for diagnosis, requiring exploratory laparotomy in order to identify the source of bleeding. 10

 However, preferably in a patient with a stable hemodynamic status, endovascular therapy remains the first-line option for known arterial bleeding and is considered a safe and effective modality to treat visceral pseudoaneurysms. 17 ^,^ 18

 One treatment option is to proceed with occlusion of the artery. 19 Several materials are available that can promote artery occlusion, such as coils, glue, detachable plugs, thrombin, Gelfoam, detachable balloons, and copolymer embolization. 19 ^,^ 20

 Coil embolization is a widely employed endovascular approach technique. It is advisable that the coils be delivered before and after the bleeding point; this maneuver is called “sandwich technique” or “endovascular ligation”. The main goal is to prevent the pseudoaneurysm refilling with blood through collateral pathways. 17

 Covered stents allow sealing of the point of bleeding, but these stents demand larger profiles and stiffer delivery devices than coil embolization. This technique is a good option when the pseudoaneurysm is located in arteries with large diameters and low tortuosity. 21

 In both of the cases described here, the patients exhibited the habitual clinical presentation characterized by active bleeding. However, bleeding causing respiratory symptoms only occurs in a small proportion of the cases reported in the literature. 3

 Notwithstanding, since there had been a suspicion of thoracoabdominal aneurysm in case 1, angiotomography was used to examine the chest, abdomen, and pelvis, ruling out that diagnosis and suggesting a diagnosis of pseudoaneurysm of the splenic artery. Case 2 presented with signs of active bleeding, associated with a physical examination finding of pulsating swelling in the abdomen, raising a clinical suspicion of pseudoaneurysm associated with chronic pancreatitis. 

 Digital subtraction angiography with selective catheterization of the celiac trunk offers a high degree of accuracy for diagnosis of pseudoaneurysm of the splenic artery and other branches originating from the celiac trunk and confirms the findings of other diagnostic methods. 22

 The patients in the cases described here were taken to the hemodynamic suite for “prepared” angiography, i.e. all of the endovascular equipment needed for treatment during the same surgical operation was ready. They exhibited good clinical recovery after endovascular repair of the pseudoaneurysms, were discharged from hospital from 5 to 10 days after surgery, and are currently in outpatients follow-up without intercurrent conditions. 

 On the basis of these reports, it can be concluded that there is a strong association between pseudoaneurysms of branches of the celiac trunk and pancreatitis, especially among alcoholic patients. Since this is a potentially fatal complication, it is of fundamental importance that this clinical entity is diagnosed and treated rapidly, bearing in mind that rupture results in high levels of morbidity and mortality. 

 This article also exemplifies how, with the evolution in surgeons’ technical prowess and the improvements in the materials available, endovascular surgery has become a very attractive minimally invasive option for treatment of pseudoaneurysms of the splenic and gastroduodenal arteries. 
